# Tough, adhesive biomimetic hyaluronic acid methacryloyl hydrogels for effective wound healing

**DOI:** 10.3389/fbioe.2023.1222088

**Published:** 2023-07-19

**Authors:** Zhiwei Peng, Huai Xue, Xiao Liu, Shuguang Wang, Guodong Liu, Xinghai Jia, Ziqiang Zhu, Moontarij Jahan Orvy, Yin Yang, Yunqing Wang, Dong Zhang, Lei Tong

**Affiliations:** ^1^ Department of Orthopedics, The Second Affiliated Hospital of Xuzhou Medical University, Xuzhou, China; ^2^ Xuzhou Medical University, Xuzhou, China; ^3^ Department of Emergency, Affiliated Hospital of Xuzhou Medical University, Xuzhou, China; ^4^ Department of Chemical and Petroleum Engineering, UCSI University, Kuala Lumpur, Malaysia; ^5^ Tianjin Food Safety Inspection Technology Institute, Tianjin, China; ^6^ The Wallace H. Coulter Department of Biomedical Engineering, Georgia Institute of Technology and Emory University, Atlanta, GA, United States

**Keywords:** tough hydrogels, adhesive hydrogels, hyaluronic acid methacryloyl, wound healing, antibacterial activity

## Abstract

The development of cost-effective, biocompatible soft wound dressings is highly desirable; however, conventional dressings are only designed for flat wounds, which creates difficulty with promising healing efficiency in complex practical conditions. Herein, we developed a tough, adhesive biomimetic hyaluronic acid methacryloyl hydrogels composed of chemically crosslinked hyaluronic acid methacryloyl (HAMA) network and poly(N-hydroxyethyl acrylamide) (PHEAA) network rich in multiple hydrogen bonding. Due to the multiple chemical crosslinking sites (acrylamide groups) of HAMA; the bulk HEMA/PHEAA hydrogels presented significant enhancements in mechanical properties (∼0.45 MPa) than common hyaluronic acid hydrogels (<0.1 MPa). The abundant hydrogen bonding also endowed the resultant hydrogels with extremely high adhesiveness on many nonporous substrates, including glass and biological tissues (e.g., heart, liver, lung, kidney, stomach, and muscle), with a considerable interfacial toughness of ∼1432 J m^−2^. Accordingly, since both natural hyaluronic acid derivative polymers and hydrophilic PHEAA networks are highly biocompatible, the hydrogel matrix possesses good blood compatibility (<5% of hemolysis ratio) and satisfies the general dressing requirements (>99% of cell viability). Based on these physicochemical features, we have demonstrated that this adhesive hydrogel, administered in the form of a designed patch, could be applied to wound tissue healing by promoting epithelialization, angiogenesis, and collagen deposition. We believe that our proposed biomimetic hydrogel design holds great potential for wound repair and our developed HAMA/PHEAA hydrogels are extremely promising for the next-generation tissue healings in emergency situations.

## Introduction

Hydrogels are crosslinked hydrophilic polymers with 50%–99% water content, which exhibit favorable biocompatibility and malleability to adapt to massive biological and biomedical applications (Zhang et al., 2022a; Zhang et al., 2022b; Zhang et al., 2023). Since both structural and morphological properties of functional hydrogels can be tuned through a variety of chemical modifications, their rational design and engineering have enabled new modalities for biomass delivery (e.g., biomolecules, proteins, and drugs) and tissue engineering scaffolds (Zhang et al., 2020a; Zhang et al., 2020b; Zhang et al., 2021). Compared to the general requirements of ideal wound dressings, the biocompatible hydrogels not only enable bleeding to stop and relieve the pain but also absorb excess exudate and maintain the localized moist environment for cell migration, which further enhances healing, accelerates the formation of granulation tissue, and reduces the risk of infection. More importantly, the unique crosslinking structure of hydrogels also allows for the transportation of bioactive molecules (e.g., antibiotics and pharmaceuticals) to the wound center. Such entrapped molecules inside hydrogel networks can be easily exchanged by absorbing the wound exudates during the sustainable release process after contacting hydrogels with the wound surface. For instance, many injectable extracellular matrix-like hydrogels (e.g., Chitosan and GelMA) loaded with exosomes or drugs play a significant role in the reduction of M2 macrophages polarization and *in vivo* regulation of human umbilical vein endothelial cells (Wang K. et al., 2022), which potentially promote diabetic wound healing by accelerated blood vessels regeneration and collagen deposition (Peng Y. et al., 2021). However, conventional dressings were designed for flat wounds, and there is a lack of attention paid to special wounds located on frequent movements (e.g., wrists and knees). So far, few studies have focused on investigating hydrogel dressings for such a particular condition. The major challenge in wound dressing development is that the flexibility and tissue adhesion properties of traditional hydrogel dressing are not strong enough to adapt to wounds with high-frequency motion. Furthermore, the existing hydrogel dressings are expensive, and the design is complex. These factors hamper the clinical translation of hydrogel dressings. Therefore, to develop a hydrogel dressing by materials selection or preparation process modification to meet the current need is meaningful (Yu Y. et al., 2022).

Hyaluronic acid (HA) is a clear and gooey disaccharide composed of D-glucuronic acid and N-acetyl-D-glucosamine that is naturally produced in the human body, particularly found in connective tissues, eyes, and skin. Its main function is to retain water to keep organs/tissues well-lubricated and moist. Different from other polysaccharides (e.g., starch, cellulose, chitosan, and agar), HA itself is a major component of the intra-/extracellular matrix that plays a more important role in inflammation response and tissue regeneration. However, HA is not a photosensitive material and cannot form a dense cross-linking network under UV light. Interestingly, hyaluronic acid methacryloyl (HAMA), a natural extracellular matrix (ECM) with anti-inflammatory effects, promoting cell adhesion and proliferation, can become an exciting alternative that can be widely used in tissue engineering and regenerative medicine (Yang R. et al., 2021; Yu Y. et al., 2022; Ren et al., 2022). It can rapidly form an insoluble hydrogel after exposure to UV light. Multiple studies have suggested that HAMA plays a role in facilitating wound healing by supporting the release of paracrine factors from stem cells, promoting the migration and proliferation of cells into the wound (Ying et al., 2019; Liu et al., 2020; Feng et al., 2021). HAMA is an ideal material for wound dressing, theoretically. However, the mechanical strength (e.g., adhesion and stretching performances) of HAMA hydrogel is weak, which limits its application for motion wounds. To overcome the limitations of single-network hydrogels, compounds are usually introduced to fabricate composite hydrogels. A previous study has shown this by incorporating poly(N-hydroxyethyl acrylamide) (PHEAA) into gelatin; the composite hydrogel exhibited good adhesion and stretching performances (Wang S. et al., 2022). Based on this, we hypothesized that the combined advantages of HAMA and PHEAA to design a new hydrogel dressing are of great importance for the management of wounds in movable parts.

**FIGURE 1:**
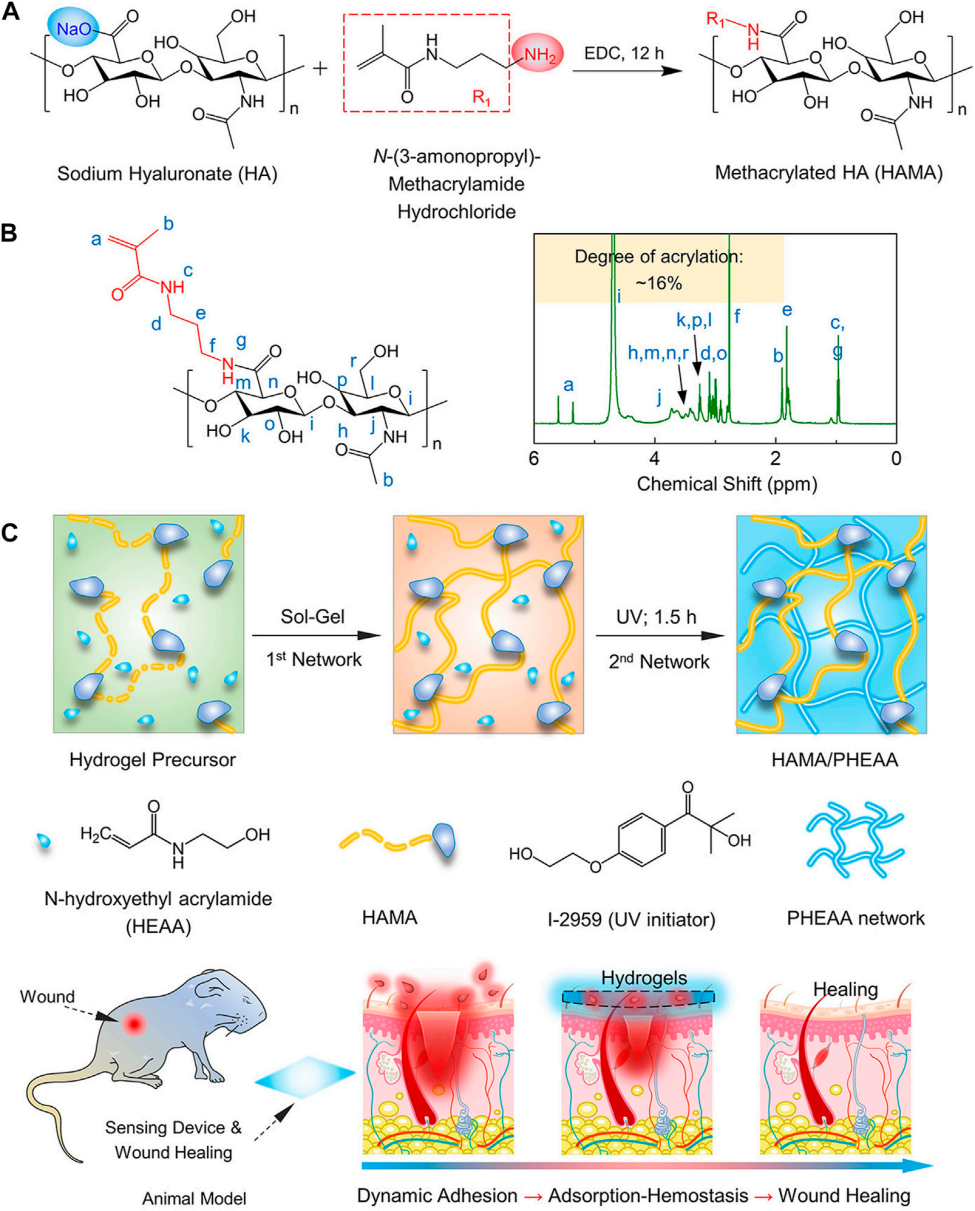
The design strategy of strong and bioactive double-network hydrogels for wound healing. **(A)** Synthesis procedure and **(B)** ^1^H-NMR spectrum of methacrylated hyaluronic acid *via* the condensation reaction between sodium hyaluronate and N-3-aminopropyl methacrylamide. **(C)** Schematic of the synthesis for HAMA/PHEAA hydrogels as wound dressings. Insets include the chemical structures of functional monomer HEAA, biomacromolecules HAMA, UV initiator I-2959, and other network structures, respectively.

Herein, we proposed and developed HAMA/PHEAA (HP) hydrogel dressings via the one-pot, two-step method (Figure 1). Specifically, HAMA was synthesized by using the condensation reaction (degree of acrylation: ∼16%) between sodium hyaluronate and N-3-aminopropyl methacrylamide in the presence of 1-ethyl-3-[3-dimethylaminopropyl]carbodiimide hydrochloride (EDC). Due to the multiple chemical crosslinking sites (acrylamide groups) of HAMA, the bulk HP hydrogels presented significant enhancements in mechanical properties after UV polymerization. We then systematically evaluated the effect of monomer concentrations on mechanical and adhesive performances to screen the optimal specimen for subsequent biological research and applications. Interestingly, both HAMA and PHEAA networks enable the control of the normal inflammatory process by serving as a signal suppressor that downregulates specific enzymatic activities due to their highly hydrophilic and biocompatible nature. Such mechanically strong, biocompatible, adhesive HP hydrogels were then designed as soft dressings that can effectively promote full-thickness wound closure. Accordingly, this hydrogel holds great potential for wound repair and is promising for application in emergency situations.

## Results and discussion

### Synthesis and mechanical characterizations of HAMA/PHEAA hydrogels

To obtain bioactive and robust hydrogels, we initially synthesized hyaluronic acid methacryloyl (HAMA) through a typical condensation reaction, effectively imparting photosensitivity to HA biomacromolecules (Figure 1A). The constructure of HAMA was analyzed using a ^1^H-NMR spectrum. The characteristic peaks of g and c in Figure 1B confirmed the successful grafting of the methacryloyl group onto the HA side chain. The degree of acrylation was estimated to be approximately 16%, calculated from the peak area ratio. The double network hydrogels were fabricated using a one-pot, two-step process (Figure 1C). In this process, the hydrogel precursor consisted of a specific amount of HEAA (0.4–1.6 g/mL), HAMA (10 mg/mL), and UV initiator I-2959 (8–24 mg/mL), mixed at 60 °C. The aqueous precursor was immediately transferred into glass molds (thickness: 1 mm). The first physical polysaccharide network was formed during the cooling process of the precursor, as the aqueous reactants gradually reduced from 60 °C to 4 °C. Subsequently, the glass molds were exposed to UV light for 2 h, initiating unsaturated double bond reactions and forming the second network. Five different HAMA/PHEAA hydrogels with varying HAMA:HEAA ratios were prepared and labeled as HP-1, HP-2, HP-3, HP-4, and HP-5, respectively (Table 1). As control groups, single-network hydrogels, namely, PHEAA and HAMA, were also chosen. The HAMA/PHEAA hydrogels, featuring strong networks and desirable bioactive properties, hold great promise for wound healing applications.

**TABLE 1:** Synthesis recipe of HAMA/PHEAA hydrogels in this work.

Hydrogels	HAMA (mg/mL)	HEAA (g/mL)	I-2959 (mg/mL)
**HP-1**	10	0.4	8
**HP-2**	10	0.7	14
**HP-3**	10	1.0	20
**HP-4**	10	1.3	26
**HP-5**	10	1.6	32
**PHEAA**	0	1.4	24
**HAMA**	60	0	0

**FIGURE 2:**
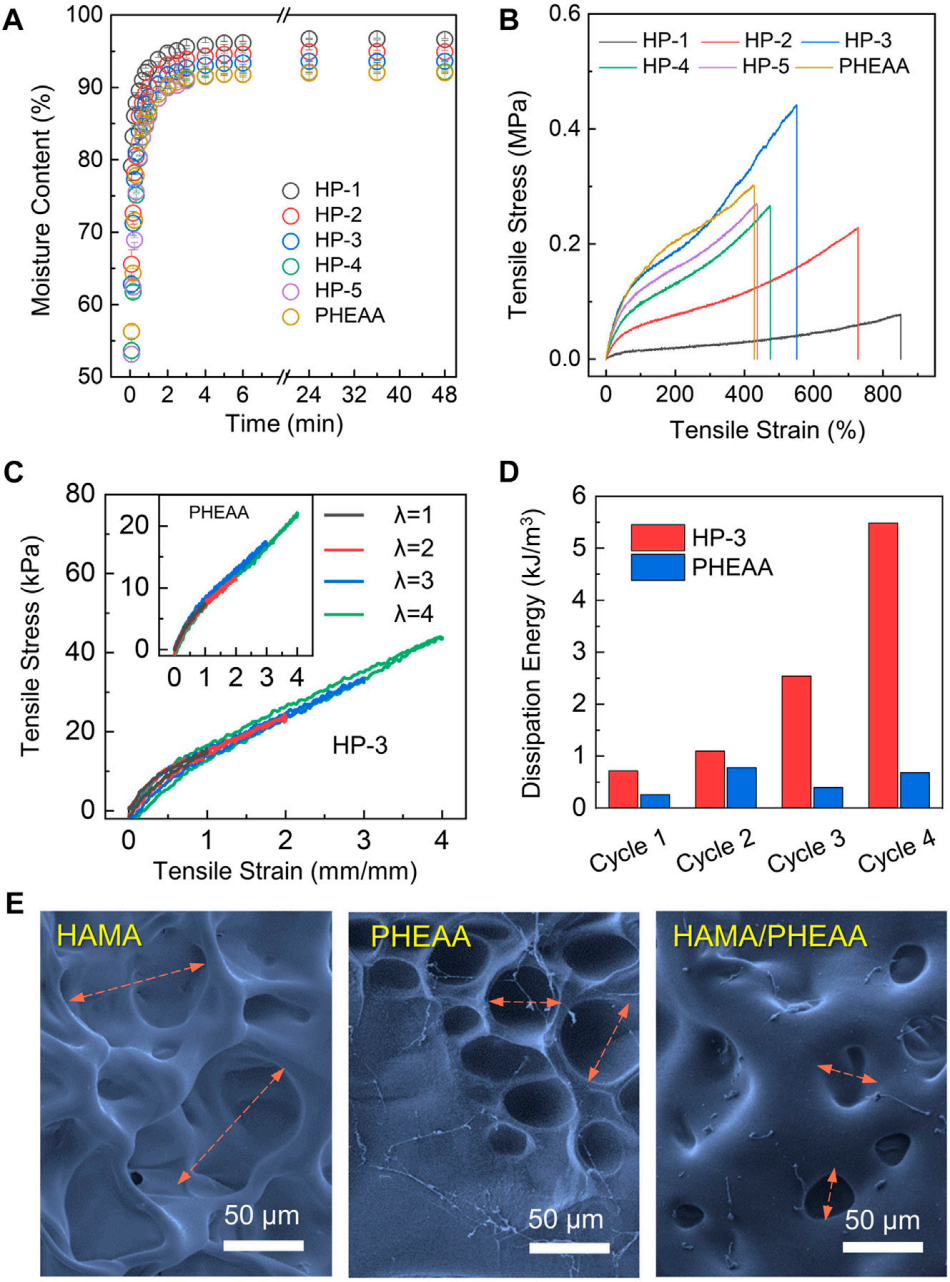
Synthesis and mechanical characterizations of HAMA/PHEAA (HP) hydrogels. **(A)** Time-dependent swelling dynamic curves of hydrogels. **(B)** The tensile stress-tensile strain of different hydrogels. **(C)** Cyclic loading-unloading curves and corresponding dissipation energies **(D)** of HP-3 and PHEAA at different tensile strains (100%, 200%, 300%, and 400%). **(E)** SEM image of hydrogels microstructure. Scale bar: 50 µm.

Upon fabricating HAMA/PHEAA hydrogels, we conducted a systematic evaluation of their physical characteristics, including swelling behavior and mechanical tensile performance. Figure 2A demonstrates that all samples exhibited rapid water absorption properties, with over 90% moisture content achieved within 3 h in a solution environment. However, the single-network PHEAA hydrogel displayed the lowest moisture content in the swollen state. Notably, as the PHEAA ratio increased, the HP hydrogels showed significantly reduced moisture content in both the initial and swelling equilibrium states. This can be primarily attributed to the incorporation of super-hydrophilic PHEAA chains within the interwoven networks. Next, we examined the mechanical properties of these five different hydrogels. As shown in Figure 2B, the tensile strain decreased with the increasing PHEAA ratio. HP-1 exhibited an impressive ability to stretch over 800%, while the pure PHEAA hydrogel could only achieve half of that strain. Moreover, the tensile strength of HP hydrogels increased with higher amounts of PHEAA incorporated into the system. Among all the samples, HP-3 demonstrated the highest break tensile strength (∼0.45 MPa) while maintaining considerable deformability (break tensile strain over 500%). Considering that flexibility and toughness are vital for wound dressing applications, hydrogels with weak mechanical strength (HP-1 and HP-2) and limited deformability (HP-5 and PHEAA) are unsuitable for potential tissue engineering applications. Consequently, we selected HP-3 as the optimized specimen for further investigation.

Energy dissipation is a crucial parameter for assessing the performance of wound patches under dynamic stretching conditions. The result of loading-unloading tests for HP-3 and PHEAA hydrogels were exhibited in Figure 2C. With four cycles, HP-3 and PHEAA both maintained relatively small hysteresis loops. The hysteresis loops of HP-3 progressively increased with strain (λ) up to four, whereas those of PHEAA decreased after the second cycle. Specifically, the dissipation energies of HP-3 were 0.714, 1.094, 2.541, and 5.485 kJ/m^3^ at the stretching strain (λ) of 1, 2, 3, and 4, respectively. The dissipation energies of PHEAA were only 0.260, 0.779, 0.397, and 0.681 kJ/m^3^ at the same state (Figure 2D). The result indicated that HP-3 was able to tolerate high stretching pressure without any breaks. To investigate the reason, we used SEM to obverse the microstructures of HAMA, PHEAA, and HP-3 hydrogels. As depicted in Figure 2E, the single-network HAMA hydrogel displayed a loose porous structure, aligning with its weakest mechanical property. The porous size of the PHEAA network was found to be less than half of the porous structures observed in HAMA, while the HP-3 hydrogels exhibited the smallest mesh among the three samples. This tight interaction between the double network components endowed HP-3 with significant mechanical strength, along with suitable energy dissipation performance.

**FIGURE 3:**
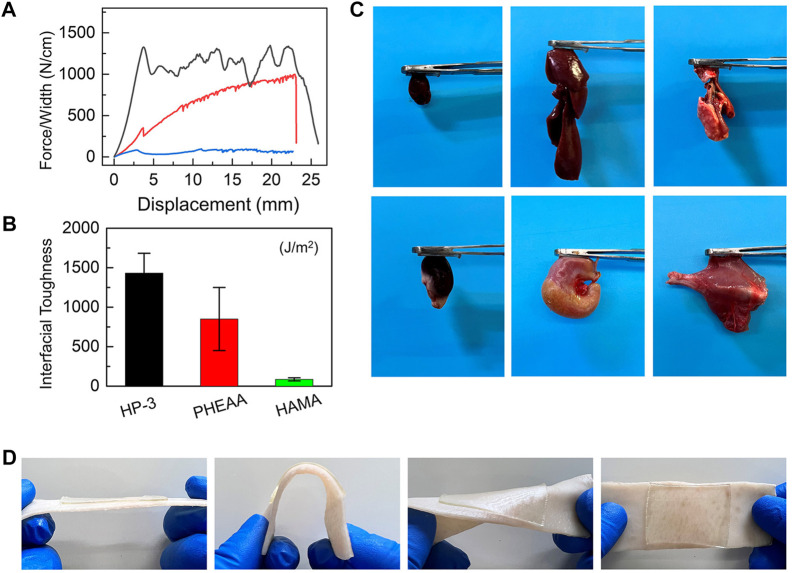
Adhesiveness of HAMA/PHEAA hydrogels. **(A)** Peeling force/width curves of HP-3, PHEAA, and HAMA on nonporous glass at a peeling rate of 50 mm min^−1^. **(B)** The interfacial toughness of three hydrogels on nonporous glass. **(C)** Photographs of the HP-3 hydrogels adhesion to different fresh organs. **(D)** Photographs of the HP-3 hydrogels adhering to porcine skin (tissues).

The strong adhesive performance on the interface is an essential index for hydrogel patches. To end this, the typical peeling force/width tests were chosen to evaluate the adhesion behavior of hydrogels. Figures 3A,B show the debonding force–displacement curves of HP-3, PHEAA, and HAMA hydrogels on nonporous glass at a peeling rate of 50 mm min^−1^. It was extremely easy to remove HAMA hydrogel from the substrate with a nearly smooth interface (<200 J m^−2^ of interfacial toughness). The peeling force/width was gradually increased with the increasing displacement of PHEAA. This indicated that the PHEAA had a homogeneous yield force, which means that a small force could lead to loss of interface and a large force is needed to remove the bulk hydrogel from the substrate. However, the yield force of HP-3 was quite larger than the peeling-off force of PHEAA, and with increased displacement, the peeling force/width was still at a large value. HP-3 exhibited strong adhesion performance during the whole process, demonstrating the tight anchoring on the interface even under accidental disturbance. The interface toughness of HP-3, PHEAA, and HAMA were 1432 J m^−2^, 850 J m^−2^, and 86 J m^−2^, respectively. HP-3 performed nearly twice and over 16 times than PHEAA and HAMA.

Next, when the wound dressing is in direct contact with the skin, strong adhesive strength is necessary for the hydrogel to stick to the skin surface and cover the wound, which can keep the wound bed clean and protect the tissue from microbial invasion. The incorporation of PHEAA into bulk hydrogel is an effective method for improving the tissue adhesion of hydrogel (Wang S. et al., 2022) because the PHEAA contains many hydrogen bonds and they can react with the functional group on tissue surfaces, and thus, endowing hydrogels with excellent adhesion strength with different substrates. The adhesive behavior of HP-3 hydrogel on different organs of rats was used to evaluate the adhesive strength. As shown in Figure 3C, the hydrogels exhibited good adhesion properties to fresh organs (e.g., heart, liver, lung, kidney, stomach, and muscle). Furthermore, we explored the hydrogel adhesiveness to porcine skin. It was found that HP-3 hydrogel remained intact and tightly adhered to the tissues after bending and distortion (Figure 3D). All of these results revealed that the HP-3 hydrogel possesses outstanding tissue-adhesive properties. The tissue-adhesive property makes it easier for the hydrogel to adhere to wounds at movable or irregular sites of the body. Therefore, HP-3 hydrogel is an ideal material for wound dressing design.

### *In vitro* antibacterial properties and biocompatibility

Infections remain common complications that can delay the healing process in wounds. Wound dressings with robust antibacterial activity are essential to prevent the contamination of external bacteria. To evaluate the antibacterial activity of the HP-3 hydrogel, tests were conducted using *Staphylococcus aureus* and *Escherichia coli*, as depicted in Supplementary Figure S1. Compared to the control group, the HP-3 hydrogels exhibited remarkable bactericidal efficacy against both *S. aureus* and *E. coli*. The number of bacterial colonies significantly decreased, and the hydrogel group achieved killing ratios of over 90% against both types of bacteria. This exceptional antibacterial property of the HP-3 hydrogels can be attributed to the unique hydration layer formed by the hydrophilic PHEAA crosslinked networks.

**FIGURE 4:**
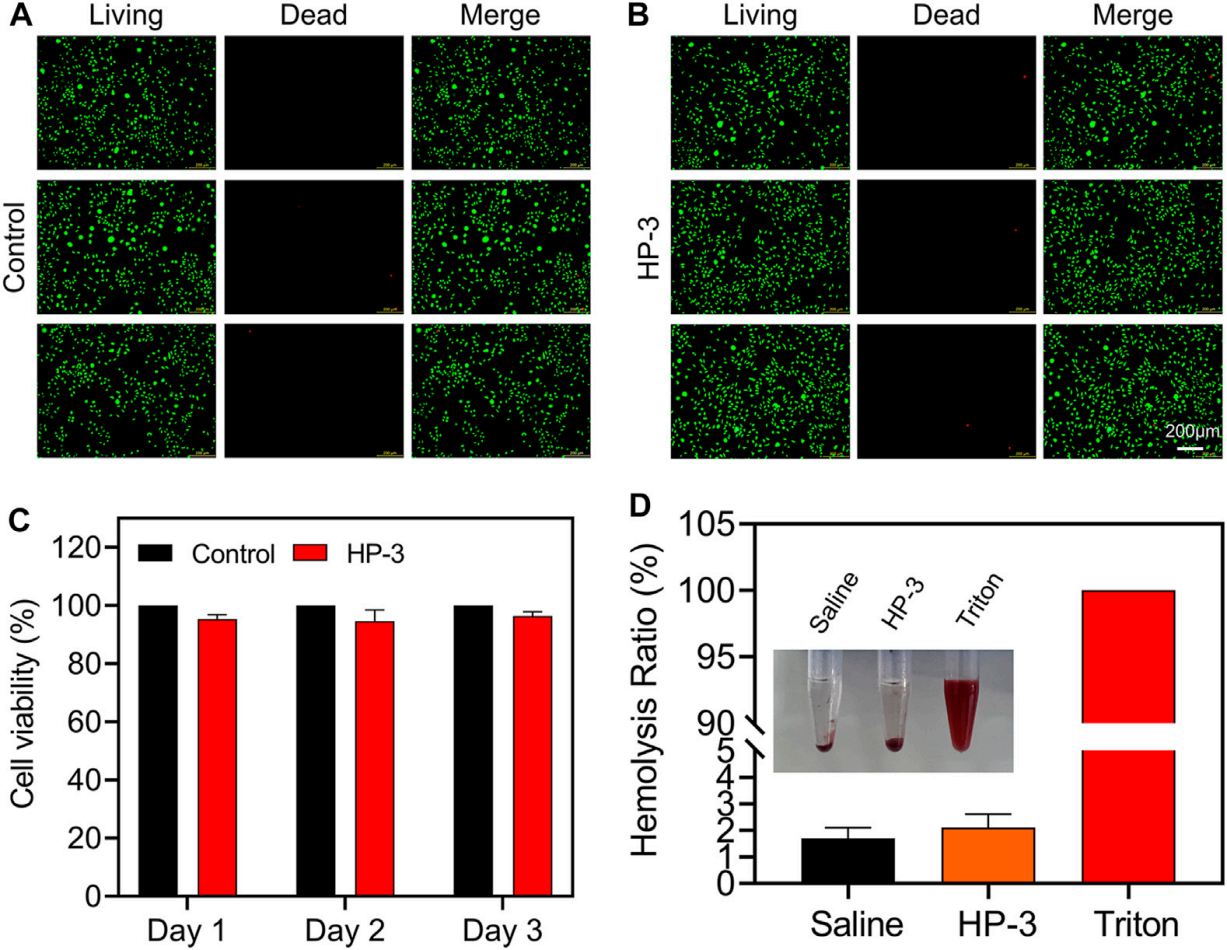
Biocompatibility of HAMA/PHEAA hydrogels. **(A,B)** Live/Dead staining images of L929 cells after incubation with or without HP-3 hydrogel for 2 days **(C)** Cell viability of L929 (n = 3). **(D)** Hemocompatibility of HP-3 hydrogel (n = 3).

Good biocompatibility and safety are vital for the animal experiment and clinical application of hydrogel dressings. Live/Dead staining and CCK-8 assay were applied to evaluate the cell viability cultivated with or without HP-3 hydrogel extracts. As shown in Figures 4A,B, after being cultured for 2 days, the morphology of living L929 cells in the hydrogel groups was similar to that of the control groups. Correspondingly, almost no dead cells were observed in both the control and hydrogel groups. Similar results were also obtained by the CCK-8 assay; the activity of L929 cells in the hydrogel groups was close to the control groups (Figure 4C). The results indicated that the cytocompatibility of the HP-3 hydrogel was satisfactory.

Skin injuries most often go along with bleeding. To be used to cover the wound, hydrogel dressings must be safe, so we further conducted a hemolytic activity assessment. The normal saline was set as the negative control group, while the Triton X-100 was set as the positive control group. As shown in Figure 4D, the supernatant was transparent in hydrogel groups and normal saline groups. In contrast, the color of the supernatant was bright red in the Triton X-100 group. Quantitative analysis of the hemocompatibility showed that the hemolysis ratios of hydrogel groups and normal saline groups were 1.8% and 2.3%, respectively. However, the Triton X-100 groups exhibited a very high hemolysis ratio (nearly 100%). The results indicated that the HP-3 hydrogel possessed good blood compatibility and met the international standard (lower than 5%) (Guo et al., 2022).

### *In vivo* wound healing study

**FIGURE 5:**
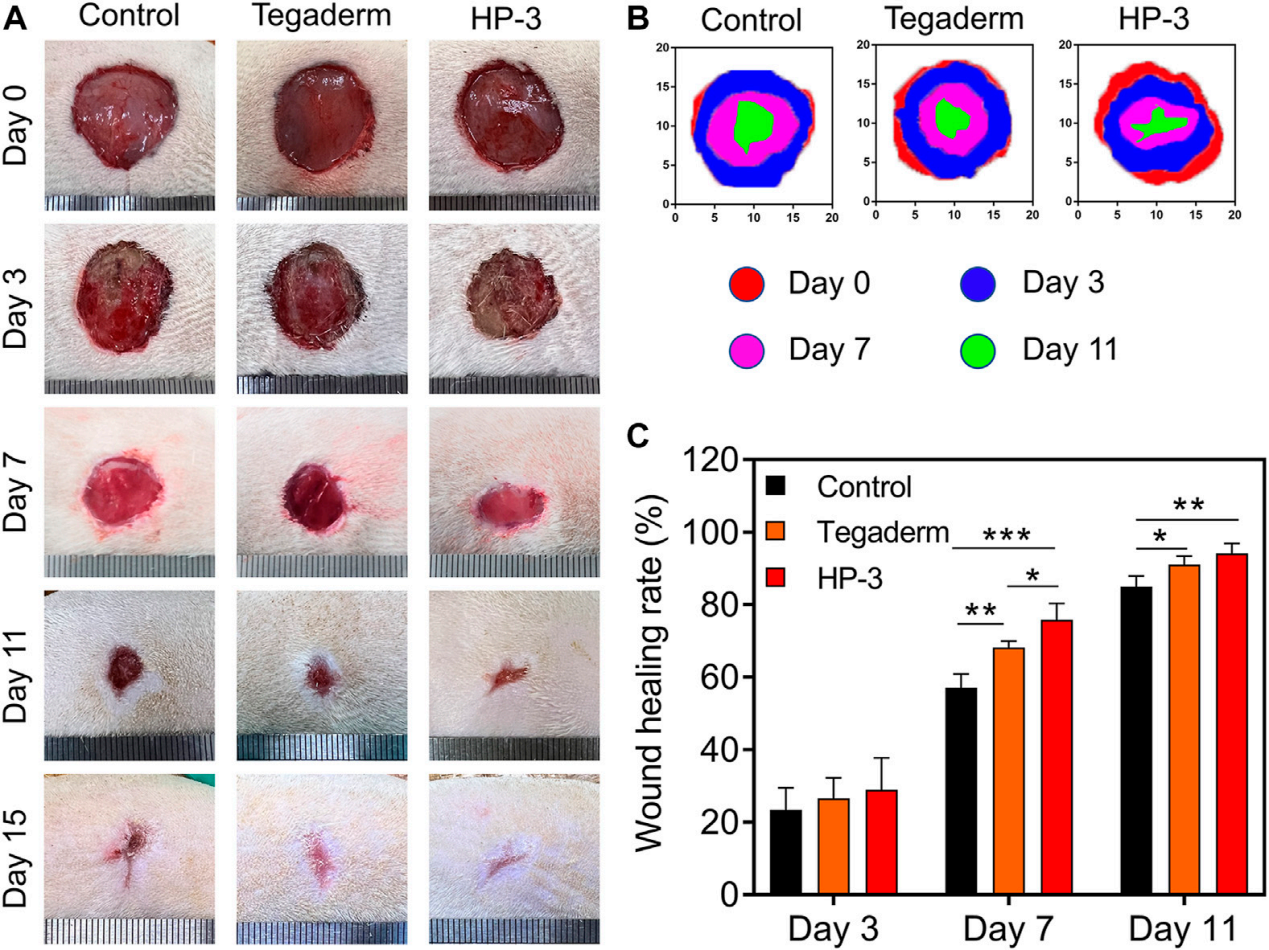
*In vivo* hemostasis and wound closure properties of HP-3 hydrogels. **(A)** Photographs and **(B)** corresponding 2D spectra of healing wounds of the control, Tegaderm, and HP-3 hydrogel groups after different treatments on days 0, 3, 7, 11, and 15, respectively. **(C)** The wound healing rate of control, Tegaderm, and HP-3 hydrogel groups on days 3, 7, and 11 (n = 5). Data represent mean ± SD; *p < 0.05, **p < 0.01, and ***p < 0.001.

To determine the wound healing effect of the HP-3 hydrogel *in vivo*, a full-thickness normal skin defect model was established. As shown in Figure 5A, the wound area of the three groups did not differ much in size in macroscopical images on postoperative day 3. The wound size of the HP-3 hydrogel groups contracted faster than the control group 7 days after surgery. When compared to commercial wound dressing, there was no obvious difference in wound size between the two groups. By postoperative day 10, the wound area of the HP-3 groups was smaller than the control and commercial product groups. The wounds healed over time. On the fifth day, the wound almost healed in the HP-3 group, while the wound was still apparent in the control group (Figure 5B). We further analyzed the wound healing rate, and the quantitative results are shown in Figure 5C. On day 7, the wound healing rate of the HP-3, control, and commercial product groups was 75%, 72%, and 65%, respectively. On day 15, the wound healing rate of the HP-3 group was approximately 98%, higher than that of the control group. The wound in the control group showed a 15% unclosed area. Hydrogels can provide a moist environment for skin regeneration and have been widely used as wound dressings (Huang et al., 2020; Yang Z. et al., 2021; Chen et al., 2021). In this study, we found that HP-3 hydrogel favored wound healing in the rat skin defect model. This can be explained as follows. The good tissue adhesion ability allows HP-3 hydrogel to tightly attach to the injured skin and provide a good microenvironment for cell proliferation and wound healing.

### Histological analysis

**FIGURE 6:**
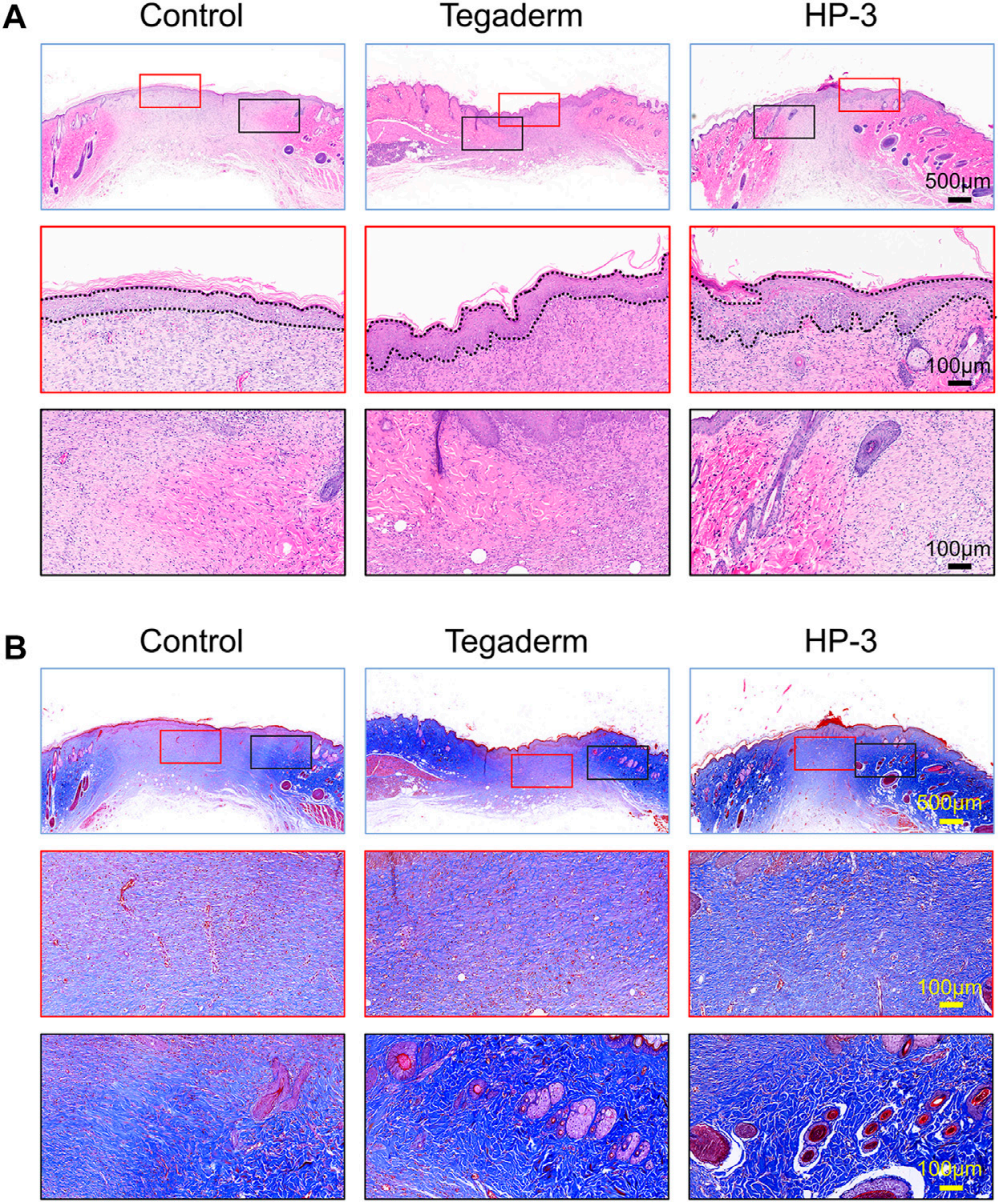
**(A)** H&E and **(B)** Masson staining images of regenerated skin tissue from different groups on day 15.

The re-epithelialization and collagen deposition are two important indicators for the evaluation of skin regeneration (Chen et al., 2020; Yu R. et al., 2022). The therapeutic effect of the HP-3 hydrogel on wounds was further evaluated with H&E and Masson staining. As shown in Figure 6A, a thicker epithelium was observed in the HP-3 hydrogel group. Meanwhile, we compared the morphology of the new collagen around the wound margin. It was observed that the newborn collagen fiber in the HP-3 hydrogel group was bold and sparsely arranged. The morphology of the newborn collagen fiber in the HP-3 hydrogel group resembled that of normal skin (Supplementary Figure S2). The collagen fiber in the control group was very small and dense, indicating weaker tissue regeneration and repair ability. Similar findings were obtained in Masson staining (Figure 6B). When compared to the control and commercial product groups, a denser collagen fiber was deposited within the wound of the hydrogel group.

**FIGURE 7:**
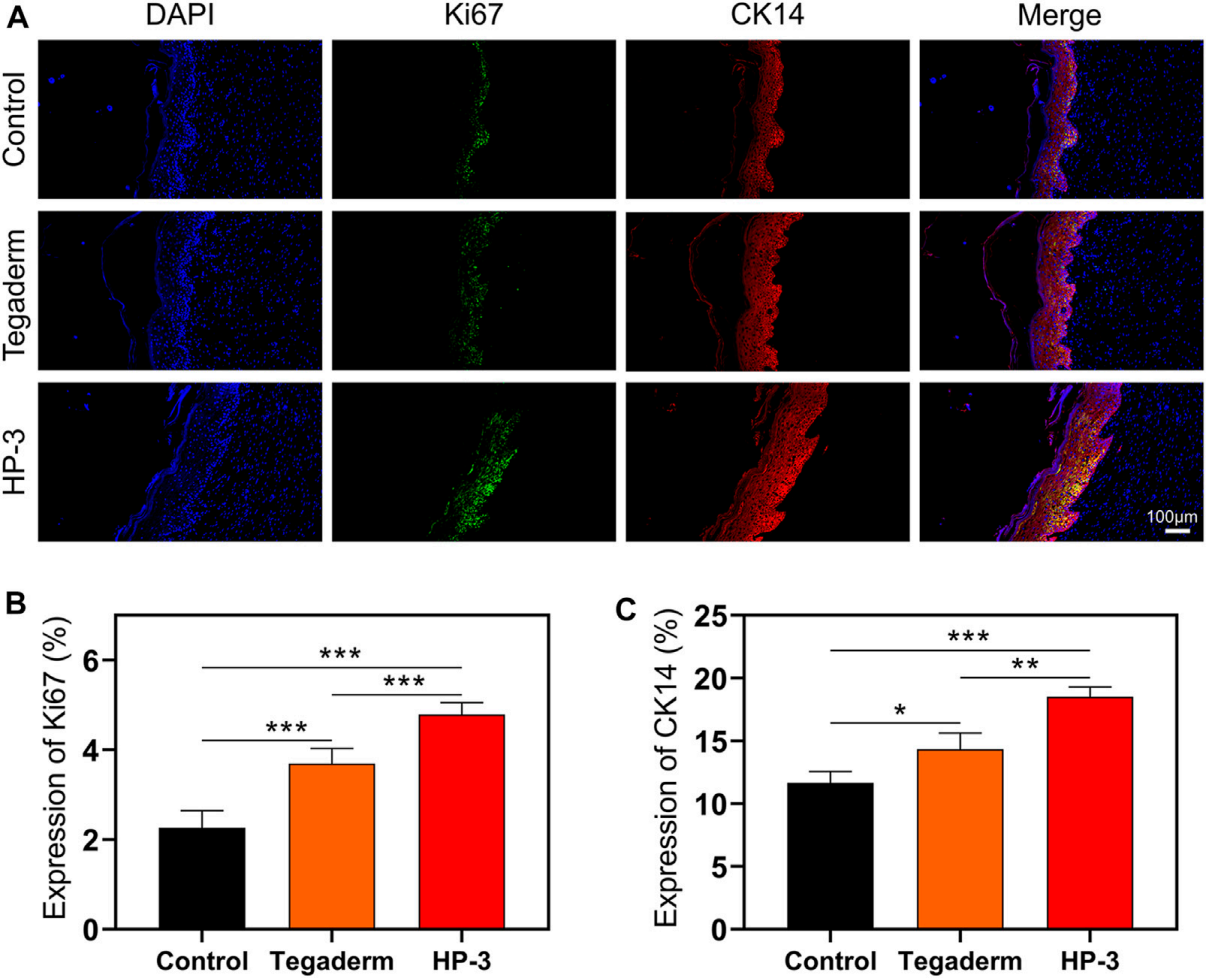
**(A)** Immunofluorescence Staining images of Ki67 and CK14. **(B)** Statistical graph of the expression of Ki67. **(C)** Statistical graph of the expression of CK14 (n = 3). Data represent mean ± SD; *p < 0.05, **p < 0.01, and ***p < 0.001.

Ki-67 is an indicator of cell proliferation and the CK14 is a common fibrin in epidermal cells (Cheng et al., 2020; Cai et al., 2022). To investigate the role of HP-3 hydrogel in tissue repair at the cellular level, we detected the protein expression of CK14 and Ki-67 in the epithelium (Figure 7A). On day 15, the hydrogel had significantly increased the density of Ki67^+^, compared to the control and commercial product groups (*p* < 0.001; Figure 7B). In addition, a significantly higher CK14 expression was found for the HP-3 group than in the control treatment and commercial groups (*p* < 0.05; Figure 7C).

**FIGURE 8:**
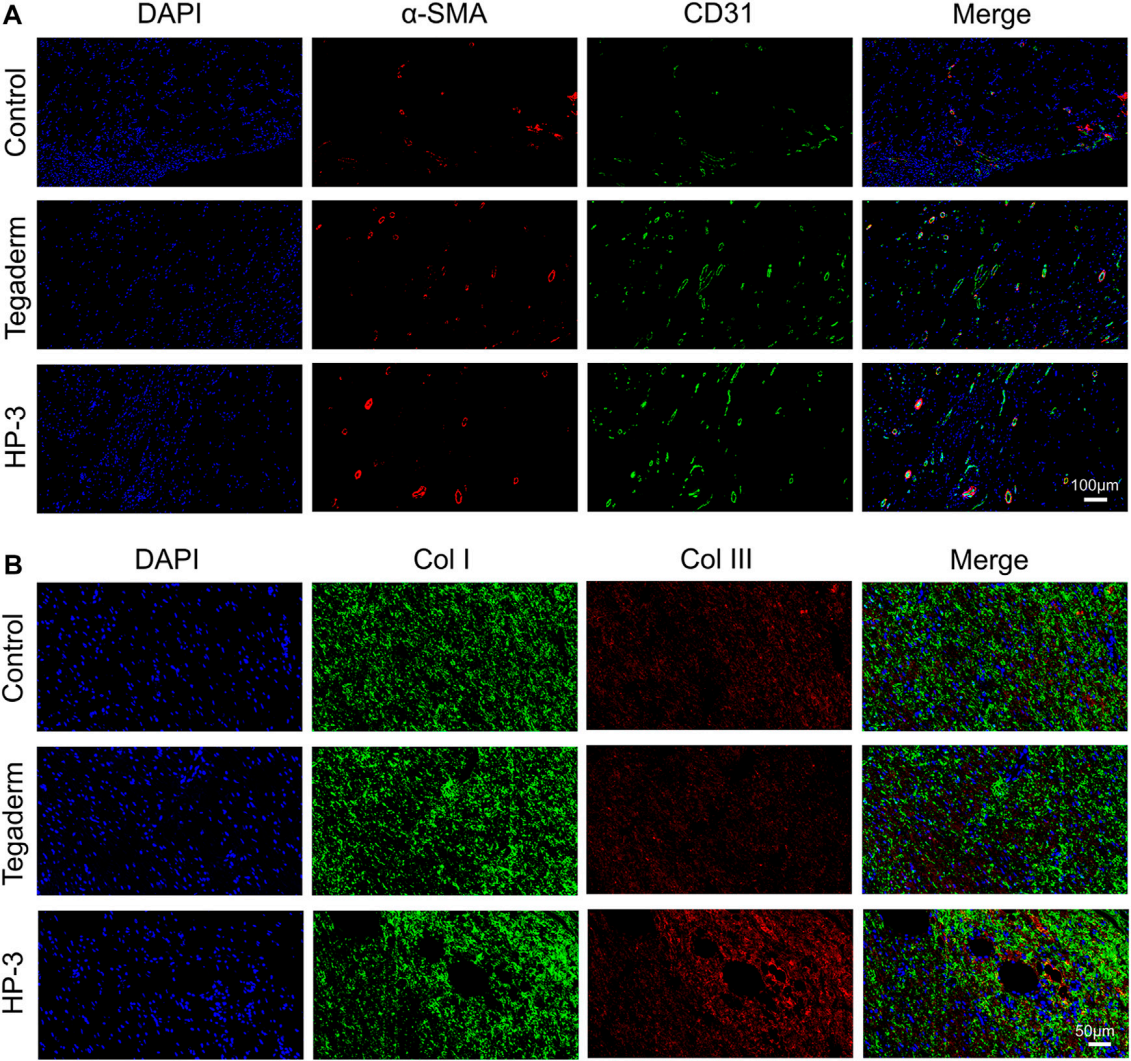
**(A)** Immunofluorescence staining images of α-SMA and CD31. **(B)** Immunofluorescence staining images of Col I and Col III.

New blood vessel formation is important for the reconstruction of tissue. Double immunofluorescence staining was applied to evaluate the regeneration of blood vessels. The results of CD31 and α-SMA staining revealed that the new blood vessel formation in the hydrogel group was significantly higher than that in the other two groups on day 15 (*p* < 0.05; Figure 8A). The new blood vessels exhibited a round shape, and there was a significant increase in the density of α-SMA and CD31 in the HP-3 hydrogel group (Supplementary Figure S3). These results suggest that the HP-3 hydrogel can facilitate angiogenesis. The remodeling of the ECM is an essential process in wound healing and collagen is an important component of the ECM (Lei et al., 2021). Collagen deposition is often used as a marker to assess the repair efficacy of wound dressing (Zhao et al., 2018; Guan et al., 2021). In order to better understand the biological mechanism of hydrogel in promoting wound healing, we performed double immunofluorescence staining of collagen to assess collagen deposition in granulation tissue. As shown in Figure 8B, wounds treated in the control group and Tegaderm showed low positive expression of both Col I and Col III. However, the HP-3 hydrogel group exhibited a notably increased expression of collagen (*p* < 0.05; Supplementary Figure S4). Compared to the collagen in the other two groups, the collagen ordered alignment in the HP-3 hydrogel groups, relatively. Col I and Col III are the two main collagen types related to ECM and wound healing. Collectively, these results indicated that HP-3 hydrogel can promote the healing of wounds *in vivo* by accelerating collagen deposition.

## Conclusion

In summary, we developed a novel HP-3 hydrogel with strong tissue adhesion properties, specifically designed for wound healing applications. This hydrogel was synthesized by proportionally mixing HAMA and PHEAA. The incorporation of biocompatible HAMA allows the dressing to mimic the extracellular matrix (ECM) of skin tissues, creating an optimal microenvironment for cell migration, proliferation, and collagen synthesis. Additionally, the hydrophilicity and mechanical stability provided by PHEAA contribute to the outstanding tissue adhesion ability of the hydrogel dressing, ensuring secure adherence to the skin. Based on these properties, the resulting hydrogel significantly accelerates wound-healing processes by promoting epithelialization, angiogenesis, and collagen deposition. Moreover, the synthetic protocol is straightforward, and the materials used are cost-effective, making the HP-3 hydrogel highly promising for clinical translation.

## Methods

### Materials

Hyaluronic acid sodium salt (HA, M_W_ = 1-2 million Da), hydroxyethyl acrylamide (HEAA), N-3-aminopropyl methacrylamide, and 1-ethyl-3-[3-dimethylaminopropyl]carbodiimide hydrochloride (EDC) were purchased from Sigma-Aldrich. The monomers removed the inhibitors first before use. All other chemicals were purchased from Aldrich and used as received without further purification. DI water was purified by a Millipore water purification system with a resistivity of 18.2 MΩ cm.

### Synthesis of HAMA

The synthesis of HAMA was followed according to the published literature with minor modifications (Park et al., 2003). For instance, 0.096 g of EDC (0.5 mmol) and 0.089 g of N-3-aminopropyl methacrylamide (0.5 mmol) were added to a solution of 0.30 mmol of hyaluronic acid (HA) in 100 mL DI water. The reaction mixture was incubated for 2 h at pH 6.5. The same amounts of EDC and N-(3-aminopropyl) methacrylamide were added after 2 h and were further incubated for another 3 h. The solution was dialyzed against 10 mM sodium chloride for 24 h and DI water for 48 h (change DI water at least four times), and finally lyophilized for 12 h to give a white cake of solid methacrylate HA (HAMA). The degree of acrylation was examined by using the ^1^H-NMR spectrum.

### Preparation of HAMA/PHEAA hydrogels

Briefly, the aqueous reactants containing a certain amount of HEAA (0.4–1.6 g/mL), HAMA (10 mg/mL), and UV initiator I-2959 (8–24 mg/mL) were thoroughly mixed in a vial at 60 °C. After the components were fully dissolved, the precursor was immediately injected into the as-prepared glass mold (thickness: 1 mm). Afterward, the precursor was placed in a 4 °C refrigerator, which underwent the cooling process to form the physical polysaccharide network. Subsequently, the hydrogel was further exposed under UV light for 2 h to complete the photoinitiated polymerization of HAMA and HEAA. All other hydrogels, such as PHEAA and HAMA single network hydrogels, were prepared using a similar protocol (see Table 1).

### Mechanical measurements

All the hydrogels were pre-cut into a dog-bone shape with a width of 3.18 mm, a gauge length of 25 mm, and a thickness of 1.0 mm. Tensile measurements were all performed on a universal tensile machine (Instron 3345, MA) with a 500 N transducer at the stretching rate of 100 mm/mm.

### Adhesiveness tests

HP hydrogel samples were synthesized and cut into cuboid shapes with a length of 15 mm, width of 5 mm, and thickness of 1 mm. Fresh organs including the heart, liver, spleen, lung, kidney, and muscle were obtained from an SD rat. The organs were washed with PBS and left to dry for the following experiments. The hydrogel samples were pasted on the surface of a steel tweezer. Then, the organs were attached to the other surface of the hydrogel samples. The steel tweezer was lifted, and the adhesion situations were recorded with a camera.

Additionally, the tissue adhesion strength of the HP hydrogel was further evaluated under dynamic conditions using porcine skin, following a methodology described in a previous study (Han et al., 2020). In summary, rectangular pieces of porcine skin were purchased and prepared by removing excess hair and fat. The HP hydrogel samples were then securely affixed to the surface of the porcine skin. The skin was subjected to bending and distortion to evaluate the tissue adhesion strength of the HP hydrogel in dynamic environments.

### Biocompatibility assay

The cytotoxicity of HP hydrogel was measured using a CCK-8 and live/dead staining *in vitro*. The HP hydrogel samples were *UV* sterilized for 1 h and soaked in fresh medium supplemented with 10% FBS (Fetal Bovine Serum) and 1% Penicillin/Streptomycin for 24 h to prepare the extracts. L929 cells were seeded in a 6-well plate and incubated with fresh medium for 24 h. Then, the medium in the control groups was replaced with fresh medium and the medium in the hydrogel groups was replaced with extracts. The cell cultivation continued for another 48 h. After the incubation time, the cell proliferation was evaluated with Calcein AM/PI Kit and observed under a fluorescence microscope (Leica, Germany). Additionally, the cytotoxicity of HP hydrogel was tested using CCK-8 after the L929 cells were cultured for 1 day, 2 days, and 3 days.

### Hemolytic activity evaluation

The hemolytic activity of the HP hydrogel was tested as described in previous literature (Peng X. et al., 2021). Whole blood was obtained from a rat and centrifugated (1,000 rpm, 10 min) to separate erythrocytes. The erythrocytes were then washed with normal saline three times and diluted to a concentration of 5%. The prepared hydrogel samples were added into the erythrocyte’s suspensions and co-incubated for 24 h at 37 °C. Triton x-100 (1%) and normal saline were used as positive and negative control, respectively. After that, the suspensions were centrifuged, and the supernatants were transferred to a new 96-well plate. The absorbance of the solution at 540 nm was detected with a microplate reader (Tecan). The hemolysis ratios were analyzed according to the following equation: Hemolysis ratio (%) = $\left(Ah-An\right)\;/\left(Ap-An\right)\times 100\%$ . *Ah*, *An*, and *Ap* were the absorbance of HP hydrogel, negative, and positive groups, respectively.

### *In vitro* antibacterial assay

The antibacterial activity of the HP-3 hydrogel was evaluated using two representative strains, including *S. aureus* (*Staphylococcus aureus*) and *E. coli* (*Escherichia coli*). For example, HP-3 hydrogel specimens were placed in a 48-well plate, and the bacterial suspension (10 (Zhang et al., 2020b) mL^-1^, 5 mL) was added onto the surface of the hydrogel. After incubating for 3 h at 37 °C, the bacterial survivors were re-suspended with sterilized PBS. As the control, bacterial suspension (5 μL,10^6^ CFU mL^−1^) in sterilized PBS was diluted to obtain a homogeneous solution. After incubation for 12 h, the colony-forming units (CFU) were counted.

### *In vivo* wound healing study

Animal experimentation was approved by the Ethical Committee of Xuzhou Medical University. All SD rats (age of 6–8 weeks and weight of 220 g) were randomly divided into three groups and eight rats per group. The three groups include the blank control group, the positive group (Tegaderm film, a commercial product), and the HP hydrogel group. The rats were anesthetized with pentobarbital sodium (40 mg/kg) *via* intraperitoneal injection. After anesthesia, the hair on the back was removed with a shaver and the skin was disinfected with betadine solution. Then, there were two 16 mm of full-thickness skin defects in the dorsal back of a rat. The skin defects were covered by Tegaderm film and HP hydrogel dressing, while the blank control group was left untreated. To evaluate the wound healing, the wounds were monitored, and pictures were taken on the 0, 3rd, 7th, 11th, and 15th days. The wound area was measured using Image J software and the wound healing ratios were calculated according to the wound area.

### Histological analysis

Rats were euthanized with high-dose pentobarbital sodium at 11 and 15 days after treatment. The tissue in the surgical area was harvested and fixed with 4% paraformaldehyde for 12 h. The samples were embedded in paraffin and cross-sectioned at 3-μm slices. Then, all slices were used for the examination of hematoxylin-eosin (HE) staining and Masson trichrome staining. Images were observed and captured with bright-field microscopy (Olympus, Japan).

For immunofluorescence staining, sections were de-paraffinized with xylene and alcohol series and were followed by rehydration. Antigen retrieval was performed using a citrate antigen repair solution. Then, the tissue sections were blocked in normal goat serum for 20 min before washing with PBS three times. After that, they were incubated with α-SMA, CD31, Col I, Col III, Ki-67, and CK14 primary antibodies at 4 °C overnight, respectively. The next day, the sections were incubated in a secondary antibody, and nuclei were counterstained with DAPI. Finally, all sections were visualized and captured with a fluorescence microscope.

### Statistical analysis

Statistical analysis was performed using SPSS (version 19.0) software. The data were expressed as mean ± SD. Student’s t-test or ANOVA was applied to determine statistical significance. *p*-value <0.05 was regarded as statistically significant.

## Data Availability

The original contributions presented in the study are included in the article/Supplementary Material, further inquiries can be directed to the corresponding authors.
