# Supplementary material for: Tough, adhesive biomimetic hyaluronic acid methacryloyl hydrogels for effective wound healing
Source: Front Bioeng Biotechnol. 2023 Jul 19;11:1222088. doi: 10.3389/fbioe.2023.1222088 (PMC10395096; doi:10.3389/fbioe.2023.1222088)
Supplement: Supplementary file 1 [file DataSheet1.docx]

**SUPPORTING INFORMATION**

**Tough, Adhesive Biomimetic Hyaluronic Acid Methacryloyl Hydrogels for Effective Wound Healing**

Zhiwei Peng ^a †^, Huai Xue ^b, c †^, Xiao Liu ^c^, Shuguang Wang ^c^, Guodong Liu ^a^, Xinghai Jia ^a^, Ziqiang Zhu ^a^, Moontarij Jahan Orvy ^d^, Yin Yang ^e^, Yunqing Wang ^a^ *, Dong Zhang ^f^ *, Lei Tong ^a^ *

^a^ Department of Orthopedics, The Second Affiliated Hospital of Xuzhou Medical University, Xuzhou 221000, China. Email: [zhuziiq@163.com](mailto:zhuziiq@163.com) (Z. Zhu); [leitongxz@163.com](mailto:leitongxz@163.com) (L. Tong)

^b^ Xuzhou Medical University, Xuzhou 221004, China.

^c^ Department of Emergency, Affiliated Hospital of Xuzhou Medical University, Xuzhou, China.

^d^ Department of Chemical and Petroleum Engineering, UCSI University, Taman Connaught, 56000 Cheras, Wilayah Persekutuan Kuala Lumpur, Malaysia.

^e^ Tianjin Food Safety Inspection Technology Institute, Tianjin 300308, China.

^f^ The Wallace H. Coulter Department of Biomedical Engineering, Georgia Institute of Technology and Emory University, Atlanta, GA 30332, USA. Email: [dzhang470@gatech.edu](mailto:dzhang470@gatech.edu) (D. Zhang)

† Z. P. and H. X. contributed equally to this work.


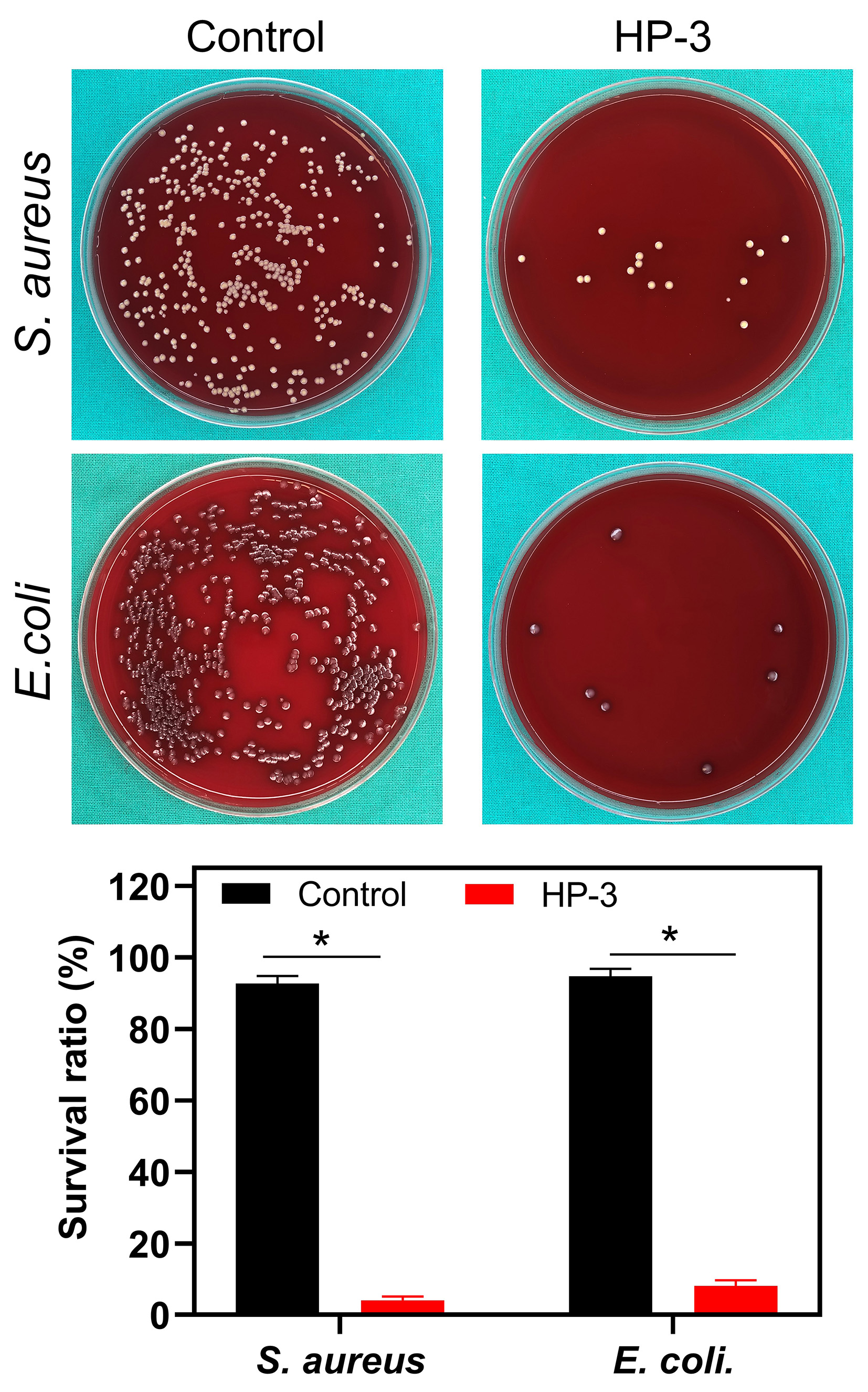


**Figure S1**. Antibacterial activity of the HP-3 hydrogels, cocultured with *S. aureus* and *E. coli*.


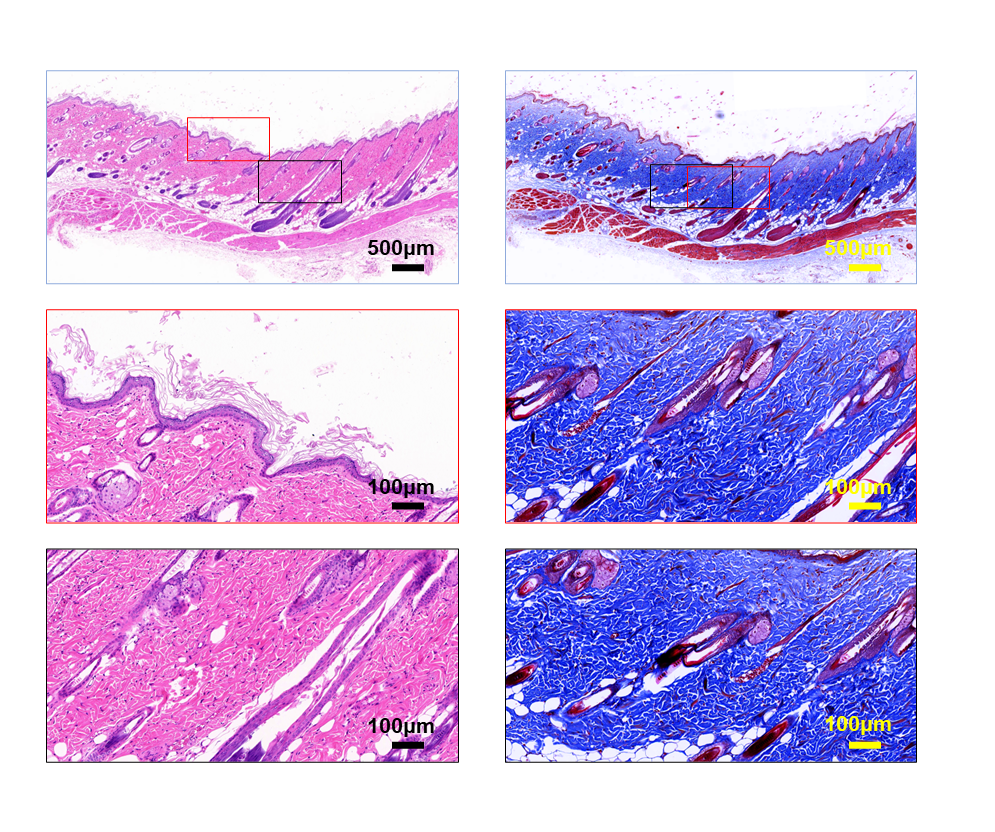


**Figure S2**. HE (Hematoxylin and Eosin) staining and Masson staining images performed on the normal skin from a healthy rat.


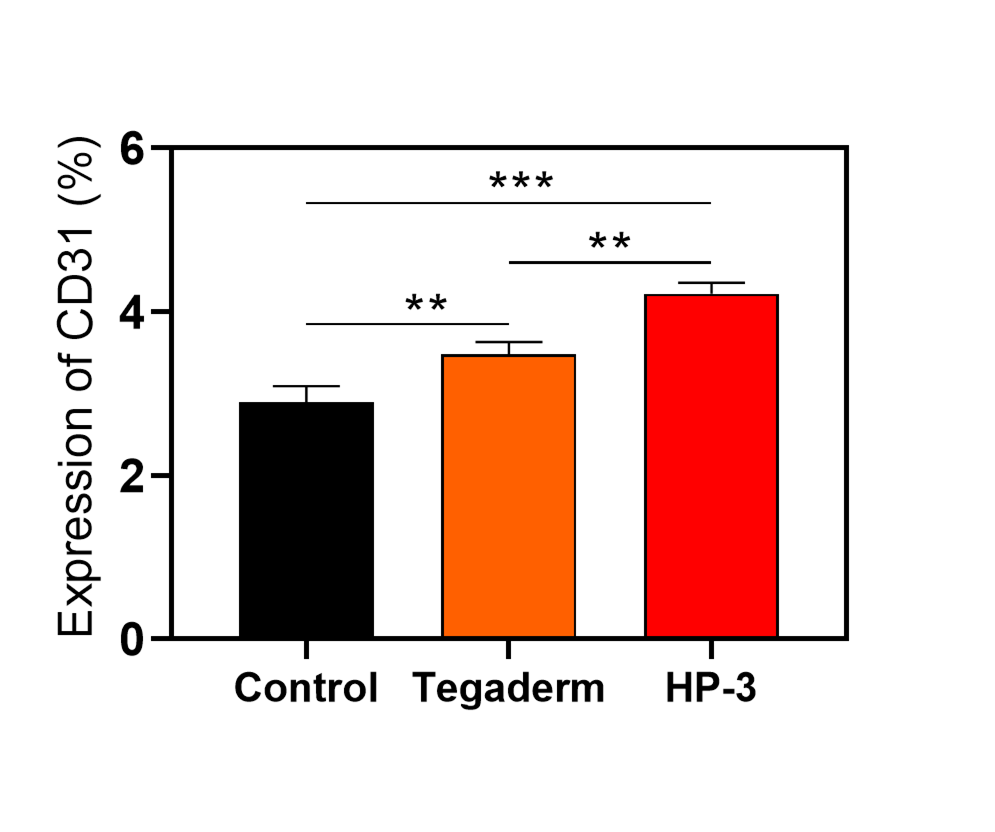


**Figure S3**. Statistical graph of the expression of CD31 (n=3). Data represent mean ± SD; *p < 0.05, **p < 0.01, ***p < 0.001.

.


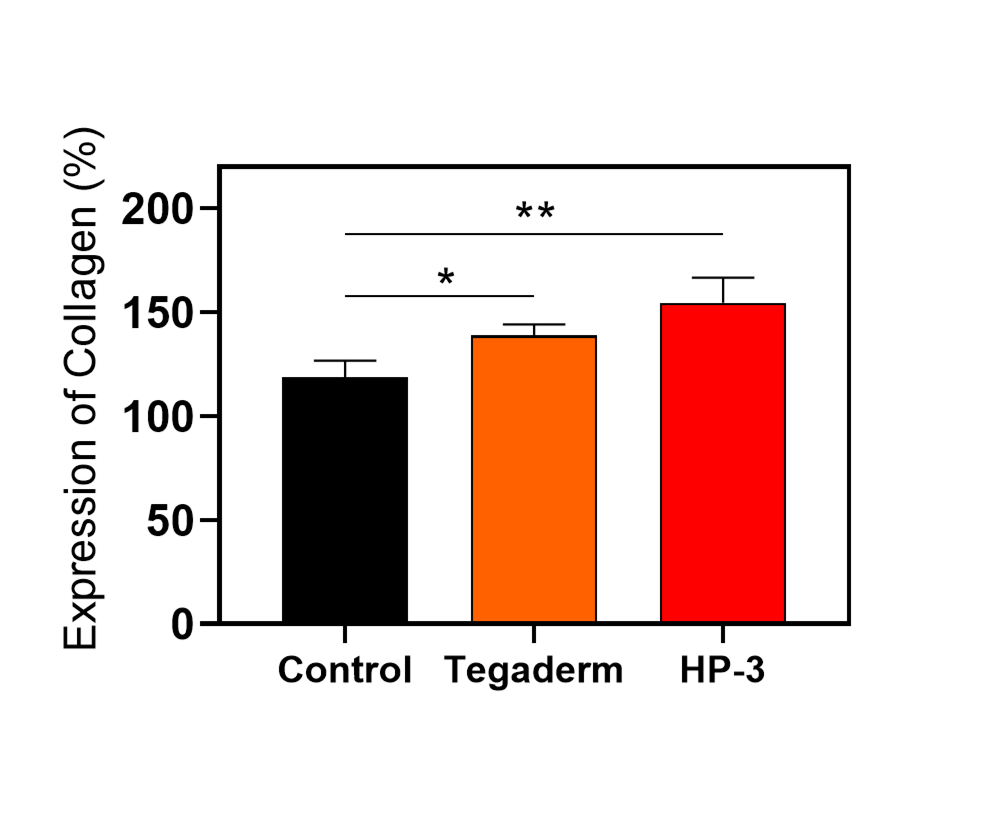


**Figure S4**. Statistical graph of the total collagen disposition (n=3). Data represent mean ± SD; *p < 0.05, **p < 0.01, ***p < 0.001.
